# Initiation of Rod Outer Segment Disc Formation Requires RDS

**DOI:** 10.1371/journal.pone.0098939

**Published:** 2014-06-04

**Authors:** Dibyendu Chakraborty, Shannon M. Conley, Muayyad R. Al-Ubaidi, Muna I. Naash

**Affiliations:** Department of Cell Biology, University of Oklahoma Health Sciences Center, Oklahoma City, Oklahoma, United States of America; Cedars-Sinai Medical Center; UCLA School of Medicine, United States of America

## Abstract

Rod outer segment (OS) morphogenesis involves assembly of flattened discs circumscribed by a hairpin-like rim, however, the role of the rim and rim proteins such as retinal degeneration slow (RDS) and its homologue rod OS membrane protein-1 (ROM-1) in this process remains unclear. Here we show that without RDS, no disc/OS formation occurs, while without rhodopsin, small OS structures form containing aligned nascent discs. In the absence of both rhodopsin and RDS, RDS-associated degeneration is slowed, and ROM-1 is stabilized and trafficked to the OS. These animals (*rho* ***^−^*** ^*/****−***^ */rds* ***^−^*** ^*/****−***^) exhibit OSs slightly better than those lacking only RDS, but still without signs of disc formation. These results clearly demonstrate that OS morphogenesis is initiated by RDS-mediated rim formation, a process ROM-1 cannot recapitulate, with subsequent disc growth mediated by rhodopsin. The critical role of RDS in this process helps explain why photoreceptors are so sensitive to varied RDS levels, and why mutations in RDS cause debilitating retinal disease.

## Introduction

Rod photoreceptor outer segments (OS) are modified primary cilia comprising precisely aligned stacks of membranous discs enclosed by the plasma membrane. Each disc has two parts, the hairpin-like rim region which circumscribes the edge of the disc and maintains the flattened morphology, and the lamellae of the disc which contains the machinery required to initiate phototransduction. The formation of these discs, and by extension the unique OS ultrastructure, requires both rim and lamellae components, however, the precise contribution of each to the process of OS morphogenesis is not well-understood. The protein and lipid components of this organelle are synthesized in the adjacent inner segment (IS) and then trafficked to their final location in the OS. Although there are many proteins found in the discs, two have been identified as critical for morphogenesis: the rim protein retinal degeneration slow (RDS, also known as peripherin-2/prds) and the lamellae protein rhodopsin. Mutations in both of these proteins are associated with a variety of blinding retinal diseases including retinitis pigmentosa (RP-rhodopsin and RDS), congenital stationary night blindness (CSNB-rhodopsin), and various forms of macular dystrophy (MD-RDS) [1], [2].

Rhodopsin is a light-sensitive visual pigment, consisting of a G-protein coupled receptor (opsin) covalently attached to the chromophore 11-*cis* retinal. Rhodopsin is found solely in rod OSs where it constitutes ∼90% of total OS membrane protein [3]. Along with its role in signal transduction, rhodopsin also plays a role in maintaining OS structure [4], [5]. Finally, rhodopsin has a critical role in the morphogenesis of the OS, due largely to the fact that most of the lipid required to build OS/disc membranes arrives in the OS as part of the rhodopsin trafficking pathway. Consistent with these functional and structural roles, in the absence of rhodopsin (*rho* ***^−^*** ^*/****−***^ mice), no rod OSs are formed and there is no rod function (as measured by electroretinogram) [6], [7]. Interestingly, heterozygous (*rho^+/^* ^***−***^) mice with ∼50% reduction in rhodopsin levels exhibit well-formed OSs which are quite similar to those found in wild-type (WT).

RDS is a tetraspanin glycoprotein restricted to the rim region of rod and cone OS discs where it forms large complexes with its non-glycosylated homologue rod outer segment membrane protein-1 (ROM-1), and together these proteins are estimated to comprise ∼4% of total OS membrane protein [8]. RDS is primarily considered a structural protein and is essential for OS morphogenesis and rim formation [9], [10]_ENREF_11. This structural role is highlighted by the observation that in a spontaneously occurring mutant mouse model which expresses no RDS protein (*rds* ***^−^*** ^*/****−***^, also known as *rd2* or *rds*), no OSs are formed [11], [12], [13]. Importantly, although RDS does not participate in phototransduction, in its absence, no photoreceptor function is present [14] in spite of the fact that the cell continues to synthesize phototransduction components [15], [16].

The process of rod OS morphogenesis is complex and at least two different proposed models exist to explain it [17], [18]. We are primarily interested in the roles of RDS/ROM-1 and rhodopsin in initiating disc formation and regulating disc size. Previous evidence has suggested that the ratio of these components is critical [19], [20], [21], [22]. For example, over-expression of rhodopsin (in the Bouse transgenic animal) leads to retinal degeneration and abnormally large OS discs [19], [20], while expression of 50% of rhodopsin (in the *rho^+/^* ^***−***^) results in OSs that are virtually normal [21], [22], [23]. In contrast, over-expression of RDS has no effects on retinal structure or function [24], while RDS haploinsufficiency (*rds^+/^* ^***−***^) results in severe OS abnormalities including the appearance of abnormally large discs, formation of sworls of disc membrane, and misalignment of discs [25]. Finally, the third component, ROM-1, must be considered. Although it is not absolutely required for OS formation, in its absence (*rom1* ***^−^*** ^*/****−***^), rod discs are abnormally large, suggesting it may play a regulatory role in OS morphogenesis [26].

Here we further explore the role of RDS/ROM-1 and rhodopsin in OS morphogenesis and retinal degeneration by generating and characterizing models expressing various amounts of these proteins. We present data to support the idea that disc formation is initiated by RDS, but not ROM-1, with growth of the disc (via addition of rhodopsin/membrane) as a secondary step.

## Materials and Methods

### Ethics statement and animal care and use

All experiments and animal maintenance were approved by the local Institutional Animal Care and Use Committee (IACUC; University of Oklahoma Health Sciences Center, Oklahoma City, OK, U.S.A., protocol number 11–128) and conformed to the guidelines on the care and use of animals adopted by the Association for Research in Vision and Ophthalmology (Rockville, MD). At the conclusion of experiments, animals were euthanized using carbon dioxide followed by cervical dislocation as approved by the OUHSC IACUC. All *Rho* ***^−^*** ^*/****−***^ mice were obtained from Dr. Janice Lem, (Tufts University, Boston, MA) while *rds* ***^−^*** ^*/****−***^ mice were originally obtained from Dr. Neeraj Agarwal (currently at the National Eye Institute, Bethesda, MD) and bred into the C57BL/6 background. Bouse transgenic mice were generated in-house and characterized previously; here we used the lowest expressing line (Bouse A) from our previous work [19]. Double heterozygote and knockout mice were generated in-house by cross-breeding. Animals were maintained in cyclic light (12 hours L:D, ∼30 lux).

### Antibodies

Primary antibodies were used as follows. Rabbit polyclonal RDS-CT, generated in-house and characterized previously [27], was used at 1∶1,000 for immunofluorescence (IF) and western blotting. Rabbit polyclonal ROM1-CT, generated in-house and characterized previously [27], was used at 1∶1,000 for western blotting. Mouse monoclonal mAB 1D4 (against rhodopsin), generously shared by Dr. Robert Molday (University of British Colombia, Vancouver, Canada) was used at 1∶1,000 for IF and western blotting. Rabbit polyclonal anti rod-opsin, generously shared by Dr. Steven Fliesler (State University of New York, Buffalo, NY), was used at 1∶1,000 for IF and 1∶100 for immunogold. Actin-HRP (Sigma, St, Louis, MO) was used at 1∶25,000 on western blotting.

### Light and electron microscopy and immunogold labeling

Tissue collection, processing, plastic-embedding, and immunogold labeling were performed as described previously [16], [28], [29]. For light microscopy, plastic embedded 0.75 µm sections were imaged with a Zeiss universal microscope. To assess ONL thickness, 40X images were captured in the central retina (along the vertical meridian) and three retinal sections containing the optic nerve head were examined (per eye) from at least three eyes per genotype. ONL thicknesses was measured using Adobe Photoshop CS5. Thin (600–800 Å) sections for transmission electron microscopy (TEM) were collected on copper 75/300 mesh grids and stained with 2% (w/v) uranyl acetate and Reynolds' lead citrate. Thin sections for immunogold were collected on nickel 75/300 mesh grids. Primary antibodies are as described above, while the secondary antibody (1∶50) was AuroProbe 10 nm gold-conjugated goat anti-rabbit IgG; (GE/Amersham, Piscataway, NJ). Sections were viewed with a JEOL 100CX electron microscope at an accelerating voltage of 60 kV.

### Immunofluorescence labeling

Eyes were harvested, dissected, fixed and embedded as previously described for paraffin sectioning (6 µm) [28]. Immunostaining was performed as described previously [28], [30] using primary antibodies as described above and anti-mouse or anti-rabbit AlexaFluor 488 or 555 conjugated secondary antibodies (Life Technologies, Grand Island, NY). Images were captured an Olympus BX-62 microscope equipped with a spinning disc confocal unit using a 40X (air, 0.9 NA) or 100X (oil, 1.4 NA) objective depending on the experiment. Images were stored and deconvolved (no neighbors paradigm) using Slidebook® version 4.2.0.3. All images shown are single planes.

### Electroretinography (ERG)

ERGs were performed as previously described [16], [27]. Following overnight dark-adaption, anesthetized, dilated mice were exposed to a strobe flash stimulus of 157 cd-s/m^2^ (using the UTAS system/BigShot ganzfeld, LKC, Gaithersburg, MD, USA) to assess scotopic ERG function. After five minutes of light adaptation at 29.03 cd/m^2^, photopic ERGs were assessed by averaging responses to 25 strobe flash stimuli at 157 cd-s/m^2^. At least 8 mice per genotype were analyzed.

### Western blot analysis

Western blot was performed as described previously [28], [31]. The retinas were homogenized on ice in solubilization buffer containing 50 mM Tris-HCl, pH 7.5, 100 mM NaCl, 5 mM EDTA, 1% Triton X-100, 0.05% SDS, 2.5% glycerol, 1.0 mM phenylmethylsulphonyl fluoride (PMSF), and 100 mg/ml N-ethyl-maleimide. SDS-PAGE and western blot were performed using standard protocols under reducing conditions (with DTT) or non-reducing conditions as indicated (hereafter referred to as reducing or non-reducing western blot). Non-reducing velocity sedimentation analysis on 5%–20% sucrose gradients was performed as described previously [31]. Experiments were repeated 3–4 times and densitometric quantification was done using the Image Lab Software (Bio-Rad, Temecula, CA).

### Statistical analysis

Quantitative assays were analyzed using two-tailed one-way ANOVA with Bonferroni's post-hoc comparisons. Significance was set at P<0.05. Data are presented as means ± SEM throughout. * P<0.05, **P<0.01, ***P<0.001.

## Results

### Increasing the rhodopsin:RDS ratio exacerbates the rate of photoreceptor degeneration

**Figure 1:**
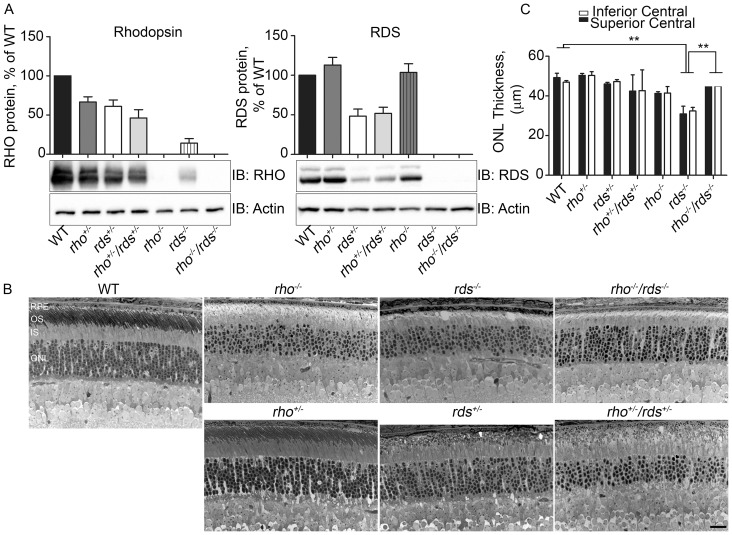
Retinal degeneration in models with varying amounts of rhodopsin and RDS. **A.** Representative western blots and quantification showing levels of RDS and rhodopsin in the indicated genotypes at P30. **B.** Representative light micrographs were captured from plastic embedded retinal sections harvested at P30 from the indicated genotypes **C.** Outer nuclear layer (ONL) thickness was measured in the central superior and central inferior retina from 3–6 eyes/genotype. Scale bar 20 µm. RPE: retinal pigment epithelium, OS: outer segment, IS: inner segment, ONL: outer nuclear layer.

To begin our assessment of the impact of the rhodopsin∶RDS ratio on OS morphogenesis, we crossed the *rds* ***^−^*** ^*/****−***^ and *rho* ***^−^*** ^*/****−***^ knockout lines to generate single (*rds^+/^* ^***−***^ and *rho^+/^* ^***−***^) and double (*rho^+/^* ^***−***^ */rds^+/^* ^***−***^) heterozygotes and double knockout (*rho^−/^* ^***−***^ */rds* ***^−^*** ^*/****−***^) lines. We also crossed the rhodopsin over-expressing transgenic mouse line (Bouse, shown to express 123% of WT rhodopsin levels [19]) onto various *rds* backgrounds. The resulting genetic rhodopsin∶RDS ratios from these crosses are summarized in the second column of **Table 1**. Because protein levels do not always follow the levels predicted by the genetic ratio (i.e. elimination of one allele does not necessarily result in 50% of protein), we measured rhodopsin and RDS levels on reducing western blots (**Fig. 1A**, **Table 1**). With the exception of the *rds^−/−^*, the rhodopsin and RDS protein levels in all the genotypes are fairly close to what would be predicted based on the genotype. However, although the *rds^−/−^* has two alleles of rhodopsin, rhodopsin protein levels are only ∼14% of WT levels, a phenomenon that has been previously observed [32], [33].

**Table 1:** 

Genotype	Rhodopsin∶RDS (Genetic Ratio)	Rhodopsin Protein** (% of WT)	RDS Protein** (% of WT)
**WT**	2∶2	100%	100%
**Rho** ***^+/−^***	1∶2	66.6%±6.7%	112.9%±9.6%
**Rds** ***^+/−^***	2∶1	61.0%±8.0%	48.3%±9.0%
**Rho** ***^+/−^*** **/** ***rds^+/−^***	1∶1	46.1%±10.67%	51.7%±7.9%
**Rho** ***^−/−^***	0∶2	0%±0%	103.5%±11.0%
**Rds** ***^−/−^***	2∶0	14.0%±5.7%	0%±0%
**Rho** ***^−/−^/rds^−/−^***	0∶0	0%±0%	0%±0%
**Bouse/WT** *	3∶2	123%	N/A
**Bouse/** ***rds^+/−^***	3∶1	N/A	N/A
**Bouse/** ***rds^−/−^***	3∶0*	N/A	N/A

* The Bouse carries two endogenous rhodopsin alleles and one transgenic allele. This line is the Bouse A as described in [19]. The Bouse line in the WT (*rds^+/+^*) background expresses 123% of WT rhodopsin levels [19].

** Protein levels are from western blots as shown in Figure 1.

**Figure 2:**
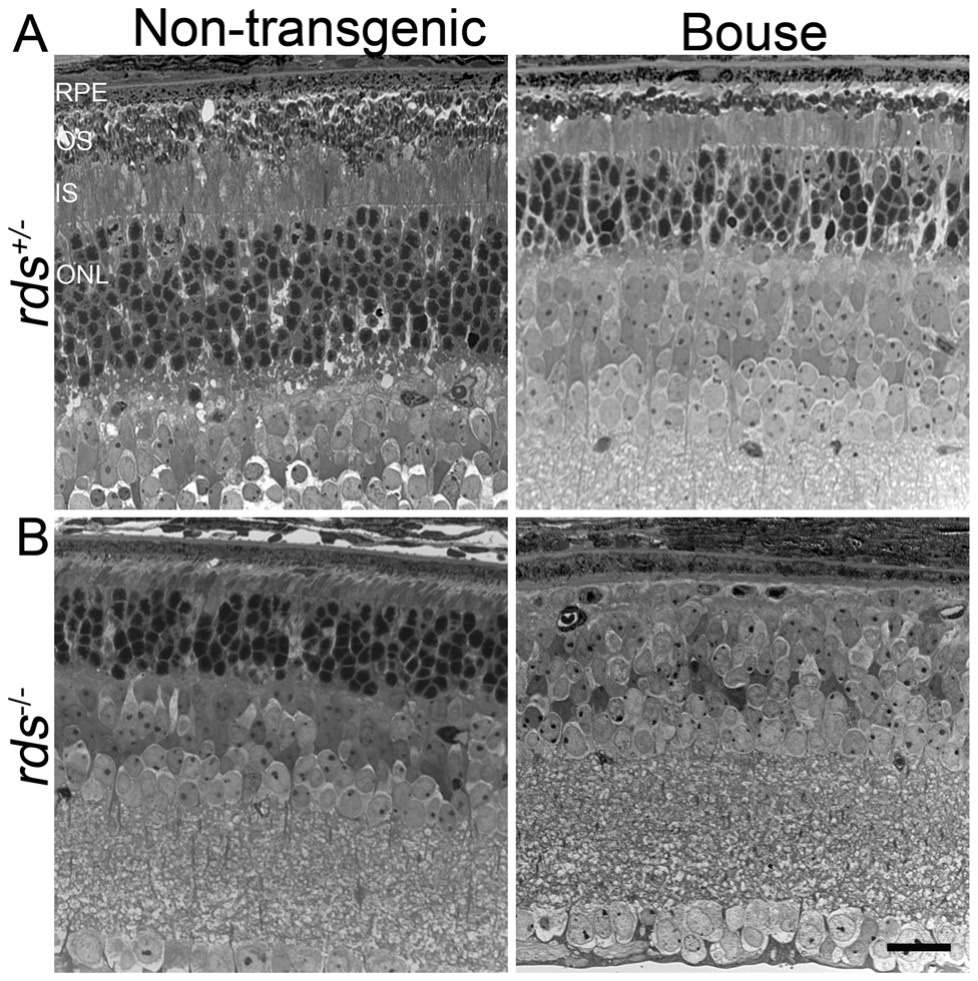
Overexpression of rhodopsin accelerates RDS-associated degeneration. Representative light micrographs were captured from plastic embedded retinal sections harvested at P30 from non-transgenic or Bouse transgenic mice in the *rds* ^+/−^ (**A**) or *rds* ^−/−^ (**B**) backgrounds. Scale bar 20 µm. RPE: retinal pigment epithelium, OS: outer segment, ISL: inner segment, ONL: outer nuclear layer.

To assess retinal degeneration, we measured the thickness of the outer nuclear layer (ONL) at P30 and observed that in both the inferior and superior retina, mean ONL thickness in the *rds^−/−^* was significantly lower than in WT (**Fig. 1B–C**). This change was ameliorated in the *rho^−/−^*/*rds^−/−^*, an observation suggesting that the elevated rhodopsin∶RDS ratio (2∶0) in the *rds^−/−^* might contribute to *rds^−/−^* associated retinal degeneration at early ages. To further assess this issue, we examined retinal structure in the Bouse/*rds^+/−^* and Bouse/*rds^−/−^* at P30. Retinal degeneration is dramatically increased in *rds^+/−^* and *rds^−/−^* mice which carry the rhodopsin over-expressing transgene: observe reduction from 8–9 rows of ONL nuclei in *rds^+/−^* animals to 5–6 rows of nuclei in Bouse/*rds^+/−^* (**Fig. 2A**), and from 6–7 rows of nuclei in *rds^−/−^* to 1 row of nuclei in Bouse/*rds^−/−^* (**Fig. 2B**).

Retinal function, as examined at P30 by ERG, showed that scotopic amplitudes in *rds^+/−^*/*rho^+/−^* mice were not significantly different from *rds^+/−^* amplitudes but were significantly less than *rho^+/−^* animals (**Fig. S1**). This suggests that RDS is the dominant factor influencing rod function in heterozygotes and showing that there is no additive effect of simultaneously reducing RDS and rhodopsin. Although scotopic amplitudes in the knockout mice were extremely low, the magnitude of the scotopic a- and b-wave amplitudes and the shape of the ERG wave form (**Fig. S1A**) were inversely proportional to the rhodopsin∶RDS ratio, that is, *rho^−/−^* (0∶2)>*rho^−/−^*/*rds^−/−^* (0∶0)>*rds^−/−^* (2∶0), an observation consistent with the idea that an increased rhodopsin∶RDS ratio results in retinal toxicity even if there is no significant phototransduction. Cones in the *rho^−/−^* are normal at early ages (**Fig. S1D–E** [34]), though photopic responses in *rho^−/−^/rds^−/−^* and *rds^−/−^* were very low.

**Figure 3:**
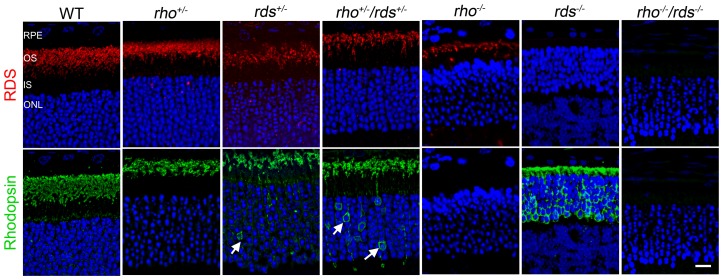
Rhodopsin mislocalization in the absence of RDS. Paraffin embedded retinal sections collected at P30 from the indicated genotypes were immunofluorescently labeled with anti-RDS (top, red) and mAb1D4 against rhodopsin (bottom, green). Nuclei are counterstained with DAPI. Shown are single planes from a confocal stack. Scale bar 20 µm. OS: outer segment, IS: inner segment, ONL: outer nuclear layer.

To further assess the defects that occur in mice with varying rhodopsin∶RDS ratios, we evaluated targeting of the two proteins to the OS in the various models. P30 retinal sections were immunofluorescently labeled with RDS (red) or rhodopsin (1D4, green) antibodies (**Fig. 3**). In the WT and *rho^+/−^*, both proteins are exclusively found in the OS, with no IS or cell body accumulation observed. Interestingly, in the *rho^−/−^*, RDS properly targets to the OS, however the reverse is not the case; rhodopsin localization is highly abnormal in the *rds^−/−^*, accumulating in the IS, and cell body. Consistent with the idea that decreasing the rhodopsin∶RDS ratio may be beneficial, this abnormal localization of rhodopsin is eliminated from most rods in the *rds^+/−^* (although a few cells continue to exhibit rhodopsin accumulation in the IS and cell body, white arrows, **Fig 3**). Finally, we observe that rhodopsin is mislocalized in a subset of photoreceptors in the *rho^+/−^*/*rds^+/−^*. Although the mislocalization of rhodopsin in the *rds^−/−^* could be due to a requirement for RDS for rhodopsin trafficking, this is unlikely as studies have suggested that the two proteins traffic separately [35]. It is more likely that the mislocalization of rhodopsin is not a trafficking defect, but rather due to the loss of OS structure in the *rds^−/−^* [11]. That is, when there is no OS in which rhodopsin can reside, it must accumulate somewhere, thus explaining its presence in the IS and cell body.

### ROM-1 traffics to the OS and forms complexes in the absence of RDS and rhodopsin

**Figure 4:**
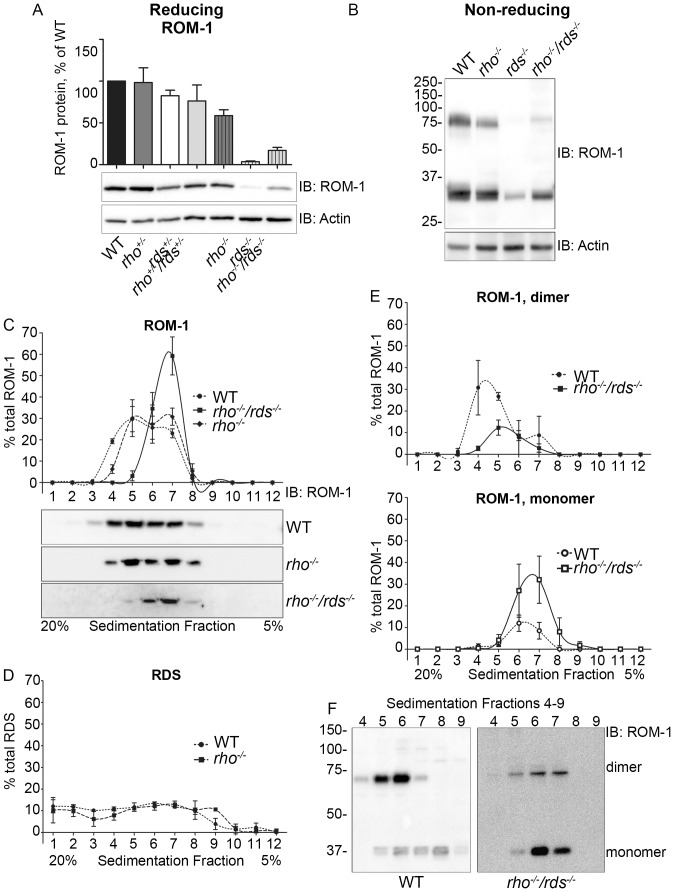
ROM-1 levels are increased in the *rho^−/−^/rds^−/−^* compared to the *rds^−/−^*. Blots shown/described in panels **A**, **C**, **D** were run under reducing conditions while those in **B**, **E**, and **F** were run under non-reducing conditions. **A**. Western blots of retinal extracts from 1-month old WT, *rho^−/−^*, *rds^−/−^* and *rho^−/−^/rds^−/−^* retinas were probed with antibodies against ROM-1 and actin (loading control). ROM-1 protein levels, normalized to actin, were densitometrically quantified from 3–6 different blots, and are presented as percent of WT. **B.** Retinal extracts from the indicated genotypes were run on non-reducing western blot and probed with antibodies for ROM-1/actin. **C**–**D.** Retinal extracts were fractionated on non-reducing sucrose gradients (5%–20%) and fractions (1–12) were subsequently assessed on reducing western blots and probed for ROM-1 (**C**) or RDS (**D**). Graphs plot percent of total ROM-1 or RDS in each fraction. **E–F.** Fractions from non-reducing sucrose gradients were assessed on non-reducing western blots and probed for ROM-1. Percent of total ROM-1 found as a dimer (disulfide linked) in each fraction is plotted in the top graph of E with percent of total ROM-1 found as a monomer in the bottom graph. **F.** Shown are representative non-reducing blots from the indicated genotype showing fractions 4–9.

Thus far our results suggest that reducing the rhodopsin∶RDS ratio may promote improved retinal health and a final piece of evidence in favor of this hypothesis can be found by examining ROM-1. Normally ROM-1 is virtually undetectable in the absence of RDS, a phenomenon we observe when looking at western blots from WT and knockout animals (**Fig. 4A**). However, we find that in the *rho^−/−^*/*rds^−/−^* retina, ROM-1 levels are increased, from an average of ∼3% of WT in the *rds^−/−^* to ∼17% of WT in the *rho^−/−^*/*rds^−/−^* (**Fig. 4A**).

In addition to suggesting that retinal health may be improved in the absence of rhodopsin, the increased levels of ROM-1 in the *rho^−/−^/rds^−/−^* give us the opportunity to study ROM-1 *in vivo* without RDS as a confounding factor, something that has heretofore been difficult. Because RDS/ROM-1 function relies on the formation of large disulfide linked complexes, one question of interest is whether disulfide bound ROM-1 complexes are observed in the absence of RDS. Non-reducing western blots probed with ROM-1 (**Fig. 4B**) showed the presence of disulfide-linked ROM-1 in the *rho^−/−^*/*rds^−/−^*, suggesting that *in vivo* ROM-1 does not require RDS for intermolecular complex assembly.

To further characterize the type of ROM-1 complexes which form in the absence of RDS, we conducted non-reducing velocity sedimentation on 5%–20% sucrose gradients. Previously, we have shown that in the WT retina, ROM-1 appears in two overlapping pools: octameric complexes are found in fractions 4–5, and core tetrameric compounds are found in fractions 6–9 (**Fig. 4C**) [31]. In contrast, RDS is found in these tetrameric and octameric pools but also forms large, higher-order oligomers which are found in fractions 1–3 and do not contain ROM-1 (graphic representation of the distribution of RDS in each fraction is shown in **Fig. 4D**). After running gradient fractions (labeled 1–12, **Fig. 4C**) on reducing western blots and probing for ROM-1, we observe that the distribution of ROM-1 is normal in both the WT and *rho^−/−^* with pronounced appearance of both octameric and tetrameric forms. This can be seen graphically as a wide almost biphasic peak in the top panel of **Fig. 4C** (which plots % of total ROM-1 in each fraction). In contrast, in the *rho^−/−^/rds^−/−^* the peak is shifted to the right, with less octameric ROM-1 and a concomitant increase in the tetrameric form. To determine whether any of these complexes contain intermolecular disulfide bonds, we next ran gradient fractions on non-reducing western blots and probed with ROM-1. In the WT, the majority of disulfide linked (i.e. dimeric) ROM-1 is found in the octameric fractions with only a small amount in the tetrameric fractions (dotted line, top panel, **Fig. 4E**, and left panel, **Fig. 4F**). Less of the ROM-1 is disulfide-linked in the *rho^−/−^/rds^−/−^* than the WT (solid line, top panel, **Fig. 4E**) but it is detected. This dimer peak is right-shifted, supporting data from the reducing blots (**Fig. 4C**) showing that less ROM-1 is present as octamer in the *rho^−/−^/rds^−/−^* than in the WT. Non-disulfide linked ROM-1 (i.e. monomeric) is found almost exclusively in tetrameric fractions in both the WT and *rho^−/−^/rds^−/−^* (bottom panel, **Fig. 4E**, and **Fig. 4F**). In sum, these data indicate that in the absence of RDS, ROM-1 forms covalent and non-covalently linked tetramers, and only a very small amount of covalently linked octamer.

**Figure 5:**
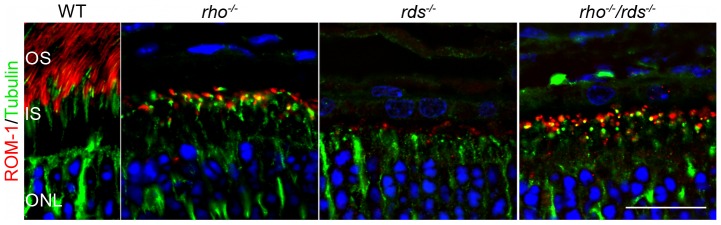
ROM-1 traffics to the OS in the absence of RDS. P30 frozen retinal sections were labeled with antibodies against ROM-1 (red) and acetylated alpha-tubulin (green). Nuclei are counterstained with DAPI. Scale bar 20 µm. OS: outer segments, IS: inner segments, ONL: outer nuclear layer.

To determine whether ROM-1 can traffic to the OS in the absence of RDS in the *rho^−/−^/rds^−/−^* photoreceptors, we conducted immunofluorescence labeling for ROM-1 (red) and acetylated alpha tubulin (a microtubule marker, green **Fig. 5**). Interestingly, we observe that ROM-1 is capable of targeting to the OS (i.e. past the IS/axoneme), observe small area of co-localization (yellow) at the base of the OS and then substantial ROM-1 past this region (red) in the WT as well as the *rho^−/−^* and the *rho^−/−^*/*rds^−/−^*. This suggests that ROM-1 does not need RDS for trafficking. These intriguing data suggest that the role of ROM-1 and the processes that govern OS targeting warrant further exploration.

### Rhodopsin is not required for the initiation of OS morphogenesis

In addition to the issue of retinal degeneration (i.e. photoreceptor cell loss), of particular interest is the role of RDS and rhodopsin (primary components of the disc rim and lamellae, respectively) in OS morphogenesis. We therefore conducted ultrastructural examination of OSs in these models at postnatal day 30. No apparent difference in gross OS ultrastructure was observed in the *rho^+/−^* or WT retinas. Consistent with previous observations [21], *rho^+/−^* contained nicely stacked and properly aligned discs (**Fig. S2**), and although it has been reported that the OSs in this genotype are slightly shorter than WT [21], we did not quantify this parameter. No rod OSs were observed at this magnification in the *rho^−/−^*, *rds^−/−^* and *rho^−/−^/rds^−/−^*, although cone OSs can be seen in the *rho^−/−^* (arrow, **Fig. S2**). OS structure in the *rho^+/−^*/*rds^+/−^* was grossly similar to the *rds^+/−^*, exhibiting membranous whorls. Interestingly we occasionally observed OSs with improved structure (arrow, **Fig. S2**), specifically improved disc packing and alignment, in the *rho^+/−^*/*rds^+/−^* compared to the *rds^+/−^* which could be due to the more favorable rhodopsin∶RDS ratio in the *rho^+/−^*/*rds^+/−^* model vs. the *rds^+/−^* (1∶1 vs. 2∶1).

**Figure 6:**
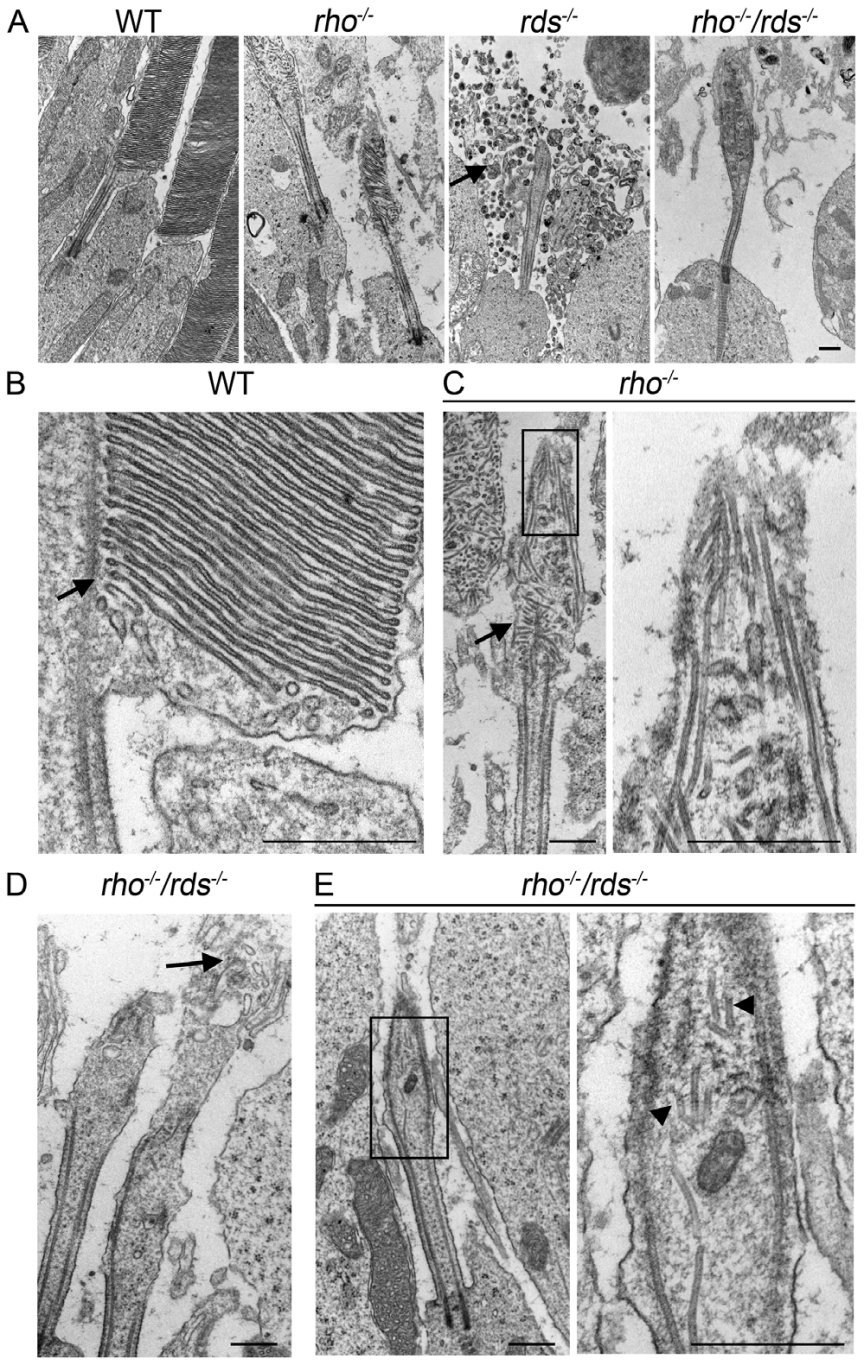
*Rho^−/−^* and *rho^−/−^/rds^−/−^* exhibit better attempts at OS formation than the *rds^−/−^*. Shown are representative high magnification TEM from the indicated genotypes (collected at P30). **A.** TEMs from each genotype, arrow indicates accumulation of vesicles outside the connecting cilium in the *rds^−/−^*. **B.** WT OS. Arrow indicates alignment of newly forming discs with axoneme. **C.** Higher magnification of OSs in the *rho^−/−^*. Arrow indicates alignment of newly forming disc-like structures with axoneme. Boxed area is shown at higher magnification on right. **D–E**. Variation in OS structure in the *rho^−/−^/rds^−/−^*. Arrow (**D**) indicates open vesicular structures in the OS. Arrowheads (**E**) indicate very small flattened membranous structures. Boxed area in **E** is shown at higher magnification on right. Images were captured at 15,000X (**A**), 25,000X (**C-left, D, E-left**) 100,000X (**B, C/E-right**). All scale bars are 500 nm.

To further assess nascent OS morphogenesis in the different models, we next examined OS ultrastructure at higher magnification (**Fig. 6**). At this level, we observe attempts at OS formation in the *rho^−/−^* but not in the *rds^−/−^* or *rho^−/−^/rds^−/−^*. In the *rds^−/−^*, the cell terminates at the apical end of the connecting cilium with no evident intracellular manifestations of attempted disc formation although we observe accumulation of extracellular vesicles and membranous material in this model (arrow, **Fig. 6A**). More interestingly, in the rhodopsin knockout, we observe decided initiation of OS formation (**Fig. 6A, C**). The *rho^−/−^* OS is characterized by the presence of small nascent disc-like structures (arrow, **Fig. 6C**). As there is no rhodopsin in this model, these structures cannot properly be termed discs, but their pinched, flattened structure makes them likely to be primarily composed of RDS-containing rim membrane. Importantly, these structures often line up properly and are oriented perpendicular to the axoneme (arrow, **Fig. 6C**) similar to newly forming discs in the WT (arrow, **Fig. 6B**). In the more distal tip of the *rho^−/−^* OS these structures lose most of this alignment, likely due to the serious ongoing malformations in disc growth which are attributed to the absence of rhodopsin. In the *rho^−/−^/rds^−/−^* (**Fig. 6A, D–E**) and structure at the distal end of the connecting cilium is frequently, although not always, better than in the *rds^−/−^*. Often, open vesicular structures are observed inside the membranous OS sack in the *rho^−/−^/rds^−/−^* (arrows, **Fig. 6D**), and sometimes a few tiny flattened vesicular structures can be seen (arrowheads, **Fig. 6E**). These structures do not properly align with the axoneme and are never as large or as frequent as those observed in the *rho^−/−^*. The microtubules which form the photoreceptor axoneme are seen in all models, however, they appear to separate abnormally in the *rds^−/−^* (**Fig. 6A**) and *rho^−/−^/rds^−/−^* (**Fig. 6A, D–E**), following the contours of the abnormal plasma membrane sac rather than remaining parallel to one another.

**Figure 7:**
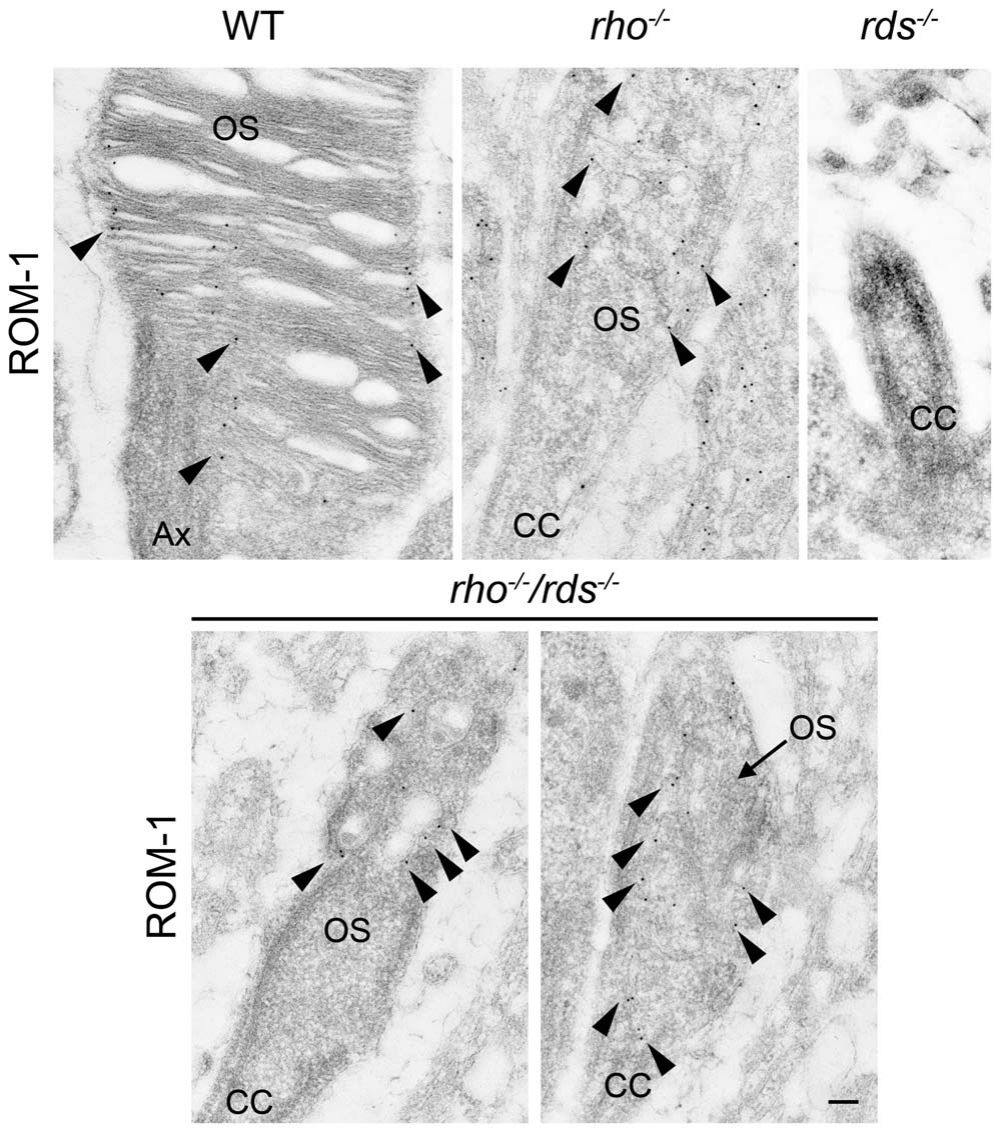
ROM-1 localizes to the nascent OS of the *rho^−/−^/rds^−/−^* by immunogold labeling. Retinal sections for the indicated genotypes underwent immunogold labeling with ROM-1 antibodies (2H5), followed by EM. Arrowheads point to ROM-1 labeling. Scale bars 500 nm. OS: outer segment, IS: inner segment, CC: connecting cilium, Ax: axoneme.

Due to their location, these small vesicular structures could contain ROM-1, so to test this, we immunogold (IG) labeled retinal sections with ROM-1 antibodies (arrowheads, **Fig. 7**). Normal rim localization of ROM-1 is observed in WT sections (**Fig. 7**), and ROM-1 is observed in the OSs in the *rho^−/−^* (**Fig. 7**). Consistent with the extremely low levels of ROM-1 in the *rds^−/−^*, no specific IG labeling is detected in this model. In contrast however, the distal tips of the *rho^−/−^/rds^−/−^* exhibit specific ROM-1 signals, in a localization that would be consistent with labeling of vesicular structures inside the OS tip (arrowheads, **Fig. 7**) like those we see on EM. These data confirm that ROM-1 is trafficked to the OS region and is on nascent disc-like structures rather than on the plasma membrane, however, ROM-1 is not sufficient to initiate disc formation.

## Discussion

We here show that ameliorating the stress associated with an abnormally high rhodopsin∶RDS ratio results in a slowing of the degeneration associated with the elimination of RDS. More importantly, we observe that eliminating rhodopsin and RDS stabilizes the rim regulatory protein ROM-1, thus highlighting the ability of this protein to form disulfide linked complexes *in vivo* without RDS and traffic independently to the OS. Finally, our data show clearly that rim formation and alignment with the axoneme is the critical first step for OS morphogenesis a process that requires RDS and cannot be recapitulated with ROM-1.

The increase in ROM-1 levels in the *rho^−/−^*/*rds^−/−^* is one of the most unexpected and exciting outcomes of this study. For many years, the role and characteristics of ROM-1 *in vivo* have been difficult to study due to the confounding presence of RDS, and the virtually undetectable levels of ROM-1 in the *rds^−/−^*. Studies in *X. laevis* using transgenic GFP-fusion constructs showed that rhodopsin and RDS both have OS targeting signals in their C-termini [36]. However, although RDS and ROM-1 have very similar structure and sequence, experiments using fusion constructs comprising the C-terminal of bovine ROM-1 and GFP demonstrated that the ROM-1 C-terminal does not have an OS targeting sequence [36] and suggested that it cannot traffic to the OS in the absence of RDS. Similarly, more recent work has shown that a single residue within the OS targeting region of RDS (valine 332) is absolutely essential for RDS targeting to the OS in mice [37], and sequence analysis shows that ROM-1 does not have an equivalent valine. Combined with the virtually undetectable levels of ROM-1 in the absence of RDS (*rds^−/−^*) these data have led to the general hypothesis that ROM-1 trafficking requires RDS. However, our data show that this is not the case. Once ROM-1 is made readily detectable (in this case by the co-elimination of rhodopsin and RDS) it traffics to the OS without RDS and does not accumulate in the IS or cell body. This observation is supported by one other report which observed small amounts of ROM-1 at the tip of the connecting cilium in the *rds^−/−^* [38]. Combined these observations suggest that not all trafficking observations from transgenic *X. laevis* studies will necessarily translate to mammals and that ROM-1 has a heretofore uncharacterized trafficking signal or trafficking pathway. We also show that *in vivo* ROM-1 does not need RDS to form core tetrameric structures. However, these tetrameric complexes do not further assemble into the larger octameric complexes which have been routinely observed to contain ROM-1 [31], [39], suggesting that assembly of ROM-1 containing octamers requires either RDS or the unique rim microenvironment created by RDS. The role of ROM-1 in rods, as well as cones (which we do not address in this study), will be a subject of great future interest.

One of the striking observations to arrive out of this work is how little aberration in the rhodopsin∶RDS ratio is needed to exacerbate degeneration. In the *rds^−/−^*, in spite of the presence of two WT rhodopsin alleles, rhodopsin levels are quite low (we measure them at 14% of WT). Yet, even this small amount of rhodopsin causes enough stress to accelerate the degeneration in the *rds^−/−^*. Similarly, in the *rds^+/−^* and *rho^+/−^/rds^+/−^* RDS levels are both very close to 50% of WT, but a reduction in rhodopsin levels from 61% of WT (in the *rds^+/−^*) to 46% of WT (in the *rho^+/−^/rds^+/−^*) is enough to promote some improvement in OS ultrastructure and disc stacking in the *rho^+/−^/rds^+/−^* compared to the *rds^+/−^*. 25% overexpression of rhodopsin in the Bouse transgenic line on the WT (*rds^+/+^*) background has previously been shown to cause retinal degeneration [19]. These observations underscore how critical it is not to have a rhodopsin∶RDS ratio that is too high. In contrast, skewing the ratio the other direction has a much less severe effect. Provided there is still a normal quantity of RDS, fairly substantial reduction in rhodopsin levels, i.e. in the *rho^+/−^* (in which we measure rhodopsin levels at 66% of WT), OSs are only slightly abnormal. Similarly, we have shown that increasing the amount of WT RDS (in the transgenic NMP model, i.e. lower rhodopsin∶RDS ratio) has no toxic effect on photoreceptors [24]. This observation hints at something that becomes more clear upon ultrastructural examination; namely that sufficient RDS is an absolute prerequisite for proper disc formation.

**Figure 8:**
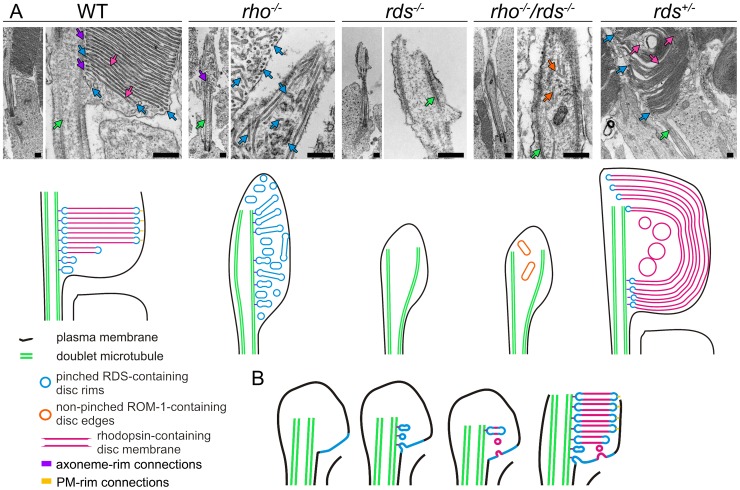
Role of RDS in OS morphogenesis. **A.** Shown are TEMs of rod OSs in the indicated genotypes (top) with diagrams representing the morphology observed in these groups underneath. Colors in the diagrams correspond to arrows in the TEMs. Scale bar, 2 µm. **B.** Hypothetical model of rod morphogenesis showing 4 steps in the formation of normal rod OSs.

Thus careful study of the abnormal OS structures found in the *rho^−/−^*, *rds* ^−/−^, and *rho^−/−^/rds^−/−^* leads to significant insight into the process of rod OS morphogenesis. Our results from **Fig. 5** **–** **7**, which are summarized pictorially in **Fig. 8A**, clearly show that initiation of disc formation and alignment with the axoneme does not require rhodopsin but requires RDS. In the absence of rhodopsin (*rho^−/−^*), disc formation is initiated, with membranous rim-containing vesicular structures lining up properly along the axoneme. In contrast, in the absence of RDS, this process is not observed. Importantly, ROM-1 cannot substitute for RDS in this process. These data enable us to propose a model of rod OS morphogenesis (**Fig. 8B**) wherein disc formation begins with small vesicles made of RDS-containing rim (blue) which are aligned and tethered to the axoneme. Opsin containing membrane then fuses to these vesicles, thus promoting their growth into mature discs. Although we do not assess it directly here, data from other studies suggest that the final size of the mature disc is likely governed by a combination of factors including: 1) the appropriate ratio of rhodopsin∶RDS [20], [22], 2) the regulatory effects of ROM-1 [26], and 3) interactions between rim proteins such as RDS and the plasma membrane [40], [41]. It is not clear what our data on the role of the rim in disc formation mean in the context of the two current models for OS morphogenesis [17], [18]. Certainly rim formation as a primary step could be a feature of either model. Although in our WT control sections (e.g. **Fig. 6B**, and **Fig. 8B**) we observe that small nascent discs are completely enclosed by the OS plasma membrane (consistent with the theory proposed in [18]), there is no way to confirm that these newly forming discs are not connected to the plasma membrane/open to the extracellular space (as in [17]) in a different plane of the section.

Finally, the accumulation of excess membranous material in the subretinal space of *rds^−/−^* animals highlights differences in the requirement for RDS between rods and cones. This material has been shown to contain rhodopsin [15] and likely buds off from the plasma membrane of rods in the *rds^−/−^* because normal OS morphogenesis is prevented by the absence of RDS. This material is not connected to the photoreceptor in any substantial portion; instead we observe connecting cilia surrounded by separate extracellular vesicles or membranes. While this material could be connected to the cell in a different imaging plane, the lack of detectable rod-based ERG signal (scotopic a-wave) makes this extremely unlikely. In contrast, we have shown that opsin containing membranes in cones lacking RDS (in the *rds^−/−^/Nrl^−/−^* [16]) remain connected to the cell (although no folded rims/lamellae are formed). Corollary studies to those presented here suggest that in contrast to rods, cone OS morphogenesis is initiated without RDS with growth of opsin containing membrane preceding rim formation [42]. These unfolded OSs contain phototransduction proteins, and are functional [16], [42]. These observations indicate that while RDS is required to form the elaborate flattened discs/lamellae in both cones and rods, the absence of RDS/rims in rods has much more drastic consequences (no OSs are formed at all, no detectable rod function), than the absence of RDS/rims in cones (abnormal lamellae-less but still functional OSs are formed).

In conclusion these data highlight the very sensitive nature of rod photoreceptors to excess opsin and suggest that abnormal accumulation of opsin outside of its normal disc milieu can contribute to ongoing degeneration in the *rds^−/−^*. The fact that amelioration of this stress by simultaneous elimination of rhodopsin and RDS stabilizes ROM-1 suggests that the *rho^−/−^*/*rds^−/−^* will be a highly useful model for studying additional properties of ROM-1. Our data also underscore the absolute requirement for RDS for photoreceptor structure and function; and serve as a reminder that a key feature of any therapeutic targeted at RDS-affected photoreceptors will have to generate sufficient protein. Finally, our data strongly support a model wherein RDS-mediated rim formation is the initiating process of rod morphogenesis with addition of rhodopsin-containing membrane occurring secondarily.

## Supporting Information

Figure S1 **Scotopic and Photopic ERG in the presence of varying quantities of rhodopsin and RDS.** Full-field scotopic (**A–C**) and photopic (**D–E**) ERG amplitudes were recorded at P30 from the indicated genotypes. **A** and **D** show representative scotopic and photopic wave forms. **B, C, E.** Shown are maximum scotopic a- and b- wave amplitudes and maximum photopic b-wave amplitudes, respectively. Data are presented as mean ± SEM from 5–7 mice per genotype.
(TIF)

Figure S2 **OS ultrastructure is slightly improved in the** ***rho^+/−^/rds^+/−^*** **vs. the** ***rds^+/−^*** **.** Shown are representative TEM images of the outer retina from eyes collected at P30 from the indicated genotypes. Arrows indicate improved OSs in the *rho^+/^* ^***−***^ */rds^+/^* ^***−***^ vs. the *rds^+/^* ^***−***^. RPE: retinal pigment epithelium, OS: outer segments. Images were captured at 3,000X, scale bar 10 µm.
(TIF)
