# Supplementary figures and images for: Initiation of Rod Outer Segment Disc Formation Requires RDS
Source: PLoS One. 2014 Jun 4;9(6):e98939. doi: 10.1371/journal.pone.0098939 (PMC4045911; doi:10.1371/journal.pone.0098939)

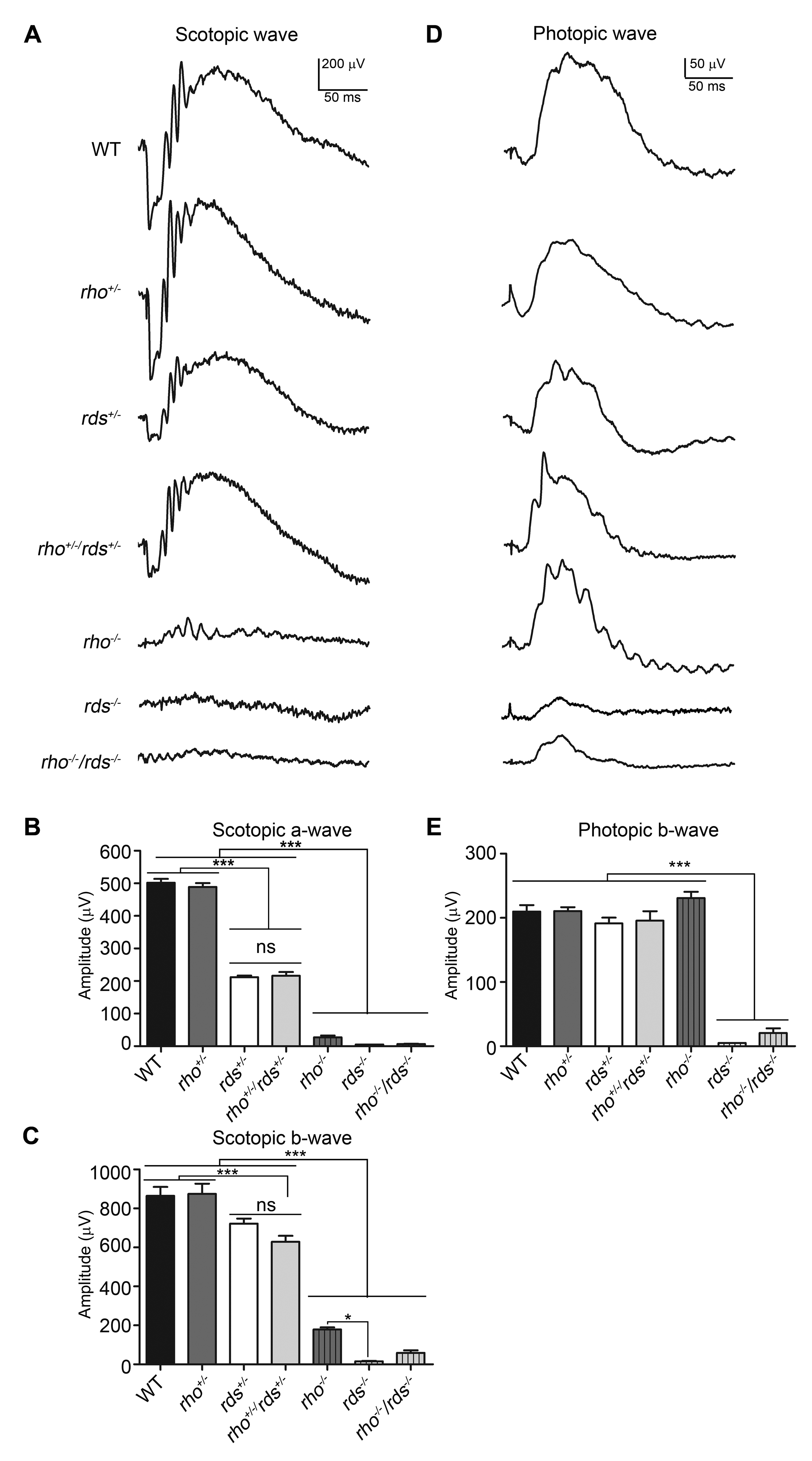

Supplement: Figure S1 — Scotopic and Photopic ERG in the presence of varying quantities of rhodopsin and RDS. Full-field scotopic (A–C) and photopic (D–E) ERG amplitudes were recorded at P30 from the indicated genotypes. A and D show representative scotopic and photopic wave forms. B, C, E. Shown are maximum scotopic a- and b- wave amplitudes and maximum photopic b-wave amplitudes, respectively. Data are presented as mean ± SEM from 5–7 mice per genotype. (TIF) [file pone.0098939.s001.tif]

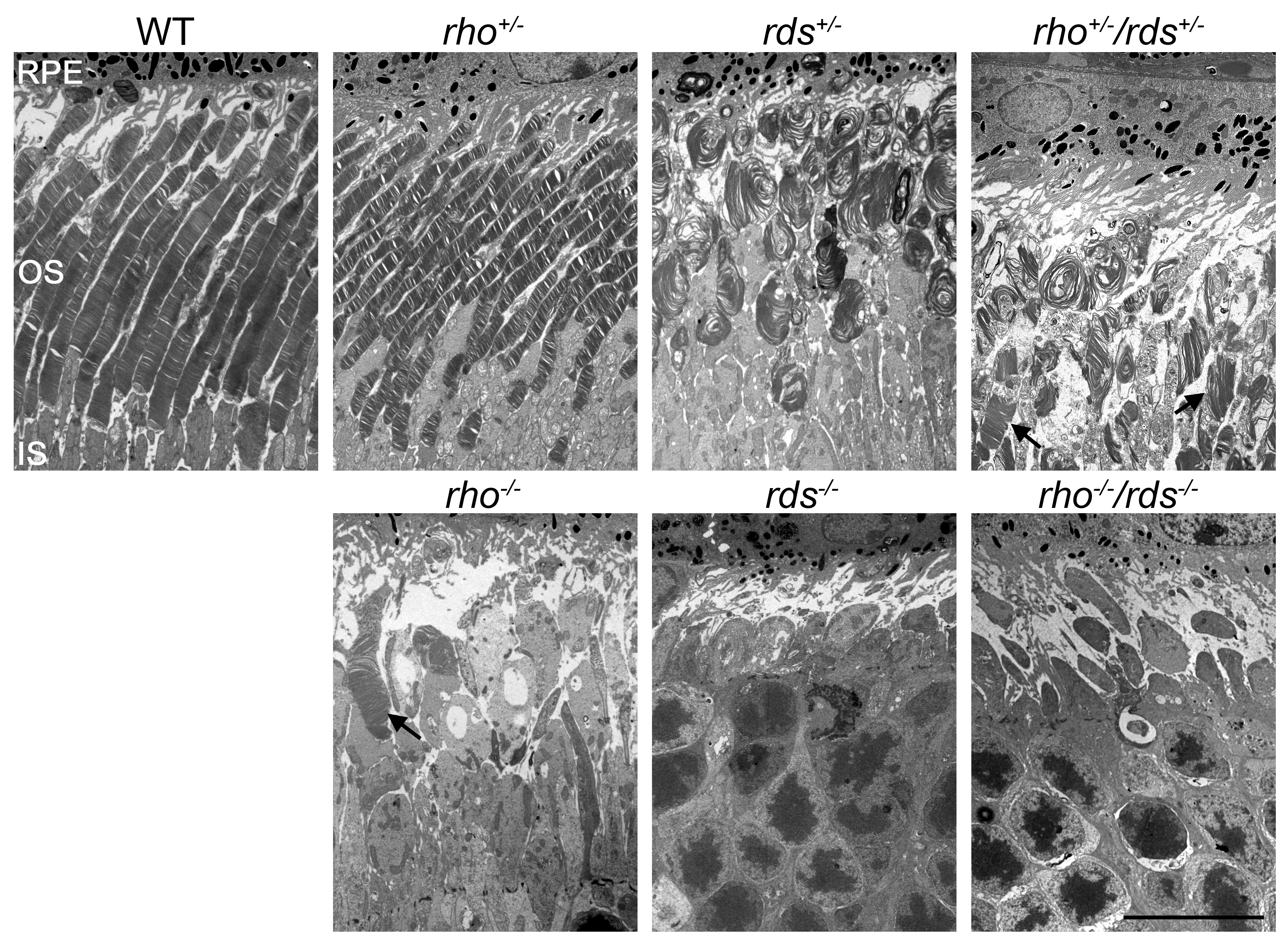

Supplement: Figure S2 — OS ultrastructure is slightly improved in the rho+/−/rds+/− vs. the rds+/− . Shown are representative TEM images of the outer retina from eyes collected at P30 from the indicated genotypes. Arrows indicate improved OSs in the rho+/ − /rds+/ − vs. the rds+/ −. RPE: retinal pigment epithelium, OS: outer segments. Images were captured at 3,000X, scale bar 10 µm. (TIF) [file pone.0098939.s002.tif]
